# Draft Genome Sequence of *Clostridium pasteurianum* NRRL B-598, a Potential Butanol or Hydrogen Producer

**DOI:** 10.1128/genomeA.00192-14

**Published:** 2014-03-20

**Authors:** Jan Kolek, Karel Sedlář, Ivo Provazník, Petra Patáková

**Affiliations:** a Department of Biotechnology, Institute of Chemical Technology, Prague, Czech Republic; b Department of Biomedical Engineering, Brno University of Technology, Brno, Czech Republic

## Abstract

We present a draft genome sequence of *Clostridium pasteurianum* NRRL B-598. This strain ferments saccharides by two-stage acetone-butanol (AB) fermentation, is oxygen tolerant, and has high hydrogen yields.

## GENOME ANNOUNCEMENT

The strain *Clostridium pasteurianum* NRRL B-598 is a spore-forming, anaerobic, mesophilic, heterofermentative, rod-shaped (young cells are motile) bacterium that differs from the recently sequenced *C. pasteurianum* DSM 525 (1), especially in its inability to utilize glycerol as a substrate and its negligible formation of ethanol and production of acetone instead of 1,3-propanediol. This strain has been used in only a few studies (2–7); however, it might be a useful platform for further genetic modification because it is not sensitive to oxygen, has versatile sugar-fermenting and proteolytic abilities, seems to be genetically stable in comparison with other clostridia, and tolerates minor changes in fermentation conditions.

Based on DNA isolation, no plasmids were present and only chromosomal DNA was obtained. For *C. pasteurianum* NRRL B-598, a single-end library was sequenced with the GS Junior System (Roche). Two sequencing runs were performed. The sequence reads from both runs were assembled with a GS De Novo Assembler 2.9 (Roche), which provided the most acceptable assembly. Annotation was added by the NCBI Prokaryotic Genome Annotation Pipeline (PGAP) (http://www.ncbi.nlm.nih.gov/genome/annotation_prok/). ProSplign (http://www.ncbi.nlm.nih.gov/sutils/static/prosplign/prosplign.html) and GeneMarkS+ (8) were used for open reading frame (ORF) detection; tRNAscan-SE (9) was used for tRNA prediction, and rRNAs were predicted by a sequence similarity search using BLAST against an RNA sequence database and/or using Infernal and Rfam models. The G+C content was calculated using the draft genome sequence. The resulting draft genome sequence of *C. pasteurianum* NRRL B-598 comprises 6,041,878 bases that are split into 138 contigs. The G+C content is 29.6%. In total, 5,547 genes were predicted by PGAP, including 5,367 protein-coding sequences (CDSs). The genome of *C. pasteurianum* NRRL B-598 is larger than that of type strain *Clostridium pasteurianum* DSM 525 (4.29 Mb) (1) as well as those of other solvent producers, e.g., *Clostridium acetobutylicum* ATCC 824 (4.13 Mb) (10) and *Clostridium acetobutylicum* DSM 1731 (11), but smaller than that of *Clostridium saccharoperbutylacetonicum* N1-4 (6.67 Mb) (12). In total, 29 rRNA and 76 tRNA genes were identified in the genome sequence.

The genome will be subjected to thorough gene mining in the near future; however, some interesting genes have already been identified, e.g., the *spo0A* gene coding for protein sporulation initiator or catalase and superoxide dismutase genes corresponding with oxygen tolerance. Also, genes involved in solvent production (*ald*, *ctfA*, *ctfB*, and *adc*) have been identified. Genes are probably clustered in operons, and all of them are highly similar to equivalent genes which were found in the genome of *Clostridium beijerinckii* NCIMB 8052.

### Nucleotide sequence accession numbers.

Data from this whole-genome shotgun project have been deposited at DDBJ/EMBL/GenBank under the accession no. AYXR00000000. Version AYXR01000000 is described in this paper.
